# Comprehensive Analysis of Aerobic Exercise-Related Genes Identifies CDCA4 That Promotes the Progression of Osteosarcoma

**DOI:** 10.3389/fgene.2021.637755

**Published:** 2021-02-03

**Authors:** Suyu Hao, Jun Zhu, Xinyue Zhang, Jingyue Qiu, Qin Xuan, Liping Ye

**Affiliations:** ^1^Shuangwu Information Technical Company Ltd., Shanghai, China; ^2^Administrative Office, Shanghai Basilica Clinic, Shanghai, China; ^3^School of Education, Hangzhou Normal University, Hangzhou, China; ^4^School of Physical Science and Engineering, East China University of Science and Technology, Shanghai, China; ^5^School of Sports Science and Engineering, East China University of Science and Technology, Shanghai, China; ^6^Department of Clinical Nursing, Minhang Hospital, Fudan University, Shanghai, China

**Keywords:** CDCA4, osteosarcoma, aerobic exercise, comprehensive analysis, progression

## Abstract

**Background:**

Exercise has a positive impact on patients with osteosarcoma, improving function, reducing disability, maintaining independence and quality of life. Exercise may also directly affect the effectiveness of cancer treatment. Cell division cycle-associated protein 4 (CDCA4) is reported to function importantly during numerous human cancers development. Nevertheless, the details toward CDCA4 function are still to be investigated.

**Methods:**

This study comprehensively analyzed the GSE74194 database and obtained aerobic exercise-related genes. Protein-protein interaction network (PPI) and Gene Ontology (GO) analysis were performed on the differentially expressed genes (DEGs). Quantitative reverse transcription polymerase chain reaction (qRT-PCR) and tumor genome atlas (TCGA) data mining were applied to measure aerobic exercise-related gene CDCA4 level in osteosarcoma tissue. We conducted lots of functional experiments to uncover CDCA4 function and its corresponding mechanism in osteosarcoma.

**Results:**

We screened a total of 547 DEGs related to aerobic exercise, of which 373 were up-regulated and 174 were down-regulated. PPI analysis revealed 90 genes that might play key roles. GO analysis showed that aerobic exercise-related DEGs were significantly enriched during the mitotic cell cycle, cell division, mitotic nuclear division and sister chromatid segregation, nuclear division, microtubule cytoskeleton organization involved protein, microtubule-based process, spindle organization, G2/M transition of mitotic cell cycle. Our results indicated that CDCA4 was increased in osteosarcoma tissues and cell lines, and its level had association with high mortality of osteosarcoma patients. Further studies revealed that absence of CDCA4 largely hindered osteosarcoma cancer cell proliferation, invasion, and migration.

**Conclusion:**

Comprehensive bioinformatics analysis improves our understanding of the underlying molecular mechanisms of aerobic exercise on osteosarcoma. This provides evidence for the effect of aerobic exercise on CDCA4 expression. Our data suggested that CDCA4 could facilitate osteosarcoma development, and gave a hint that CDCA4 was a candidate target in the treatment of osteosarcoma, aerobic exercise might help the treatment and prognosis of patients with osteosarcoma.

## Introduction

Osteosarcoma, whose morbidity ranks high in adolescence, is the most common major bone tumor (Lamoureux et al., 2007; Ottaviani and Jaffe, 2009; Taran et al., 2017). At 15–19 years old, the annual morbidity is 8–11 per million each year (Biazzo and De Paolis, 2016). At present, the details relating to oncogenesis are not conclusive. The treatment schedules for osteosarcoma involve surgery, chemotherapy and radiotherapy. However, the therapies effect is far from satisfactory and often recurs (Chen et al., 2018). Currently, 60–65% of patients treated with multi-drug neoadjuvant chemotherapy can be cured, but it also causes a number of side effects which adversely affect the patients quality of life (Picci, 2007; Serra and Hattinger, 2017). Unfortunately, this cure rate has not improved for many years, and the attempts to improve the prognosis through intensive treatment have been unsuccessful. In addition, the drugs presently included in standard chemotherapy are almost exactly the same as those used since the 1970s–80s (Serra and Hattinger, 2017). Thus, new treatments and drugs are needed to improve the overall survival rate of osteosarcoma patients. Through the systematic integration of drug combination screening, bioinformatics analysis, functional research, and correlation with clinical results, Nan et al. (2020) found that imatinib could enhance the effect of metformin on Ewing’s sarcoma by weakening the tumor hypoxia response. Also, through deep RNA sequencing, Xie et al. (2018) revealed the dynamic regulation of miRNA, lncRNAs, and mRNAs in osteosarcoma tumorigenesis and pulmonary metastasis. Despite all these advances have been made utilizing bioinformatics and science technologies to find candidate targets for osteosarcoma treatment, the outcomes of osteosarcoma patients in clinical are not significantly ameliorated (Maehara et al., 2007; Sampson et al., 2015; Bishop et al., 2016). Herein, there is an urgent need to discover available targets and explore effective treatment for osteosarcoma.

The main goal of the new field of sports oncology research is to identify the efficacy and biological mechanisms by which aerobic exercise affects the development and metastasis of cancer (Ashcraft et al., 2016). Studies have found that a combination of aerobic exercise and breathing muscle training can be included in the rehabilitation plan of non-small cell lung cancer (NSCLC) patients with poor conditions after lung resection (Messaggi-Sartor et al., 2019). It is found in breast cancer that both aerobic exercise and resistance exercise cannot prominently improve the cancer-specific quality of life of breast cancer patients undergoing chemotherapy, whereas, it can improve the constitution, body composition and chemotherapy completion rate, without causing lymphedema or major disease events (Schmidt et al., 2015; Nelson, 2016; Dieli-Conwright et al., 2018). The feasibility and use of exercise or physical exercise in the treatment of osteosarcoma and its survivors have also been discussed (Garcia et al., 2020). However, little is known about the effect of aerobic exercise on osteosarcoma and its regulation of osteosarcoma gene expression profile.

Cell division cycle-associated protein 4 (CDCA4), also called SEI-3/hematopoietic progenitor protein, has been reported to have a unique role in regulating the cell cycle (Hayashi et al., 2006; Pang et al., 2019). Presently, there are increasing researches about the impacts of CDCA4 on human diseases. For instance, Pang S et al. revealed that CDCA4 probably participated in the modulation of human triple negative breast cancer (TNBC) progression and CDCA4 might be a newly produced target in TNBC treatment field (Pang et al., 2019). Though Alderman C, et al. reported that microRNA-15a targeted CDCA4 directly and impeded malignant melanoma growth and invasiveness, the veil of CDCA4 was not fully uncovered (Alderman et al., 2016).

Here, we selected the GSE74194 dataset from the Gene Expression Omnibus (GEO) database to identify genes related to aerobic exercise. We have obtained differentially expressed genes (DEGs) between anaerobic exercise and aerobic exercise. A protein-protein interaction network (PPI) was constructed to identify key genes related to aerobic exercise. The function of the selected DEGs was further summarized by Gene Ontology (GO) annotation analysis. Kaplan-Meier plotter analyzed the survival of the CDCA4 gene. Based on The Cancer Genome Atlas (TCGA) database, the gene expression level and clinicopathological characteristics of CDCA4 were analyzed. Our data indicated that osteosarcoma tissues and cell lines highly expressed CDCA4. Functional assays demonstrated that osteosarcoma cancer cell proliferation, invasion, and migration could be dramatically inhibited upon knockdown CDCA4. Our data collectively suggested that aerobic exercise-related CDCA4 displayed as a potential carcinogene in osteosarcoma development, implying that it was a prospective marker for osteosarcoma prognosis.

## Materials and Methods

### Public Data

GSE74194^1^ was downloaded from the GEO database. The database contained transcriptome profiles of aerobic and anaerobic exercises of 10 male subjects. The Cancer Genome Atlas (TCGA)^2^ with more than 20,000 molecular characteristics of primary cancers and matched normal ones covering 33 cancer types sample, is a landmark cancer genome project. The joint efforts of the National Cancer Institute and the National Human Genome Institute initially led in 2006, bringing together researchers from different disciplines and multiple institutions.

### Analysis of GO Term Enrichment and Protein-Protein Interaction Network

The protein-protein interaction (PPI) network was applied to identify primary genes and gene modules participating in aerobic exercise. The detailed differential expressed genes (DEGs) information of PPI network derived from The Search Tool for the Retrieval of Interacting Genes (STRING) database^3^ and PPI network was established by Cytoscape software. Considering the bioconductor package “GOstats,” the significance of DEGs was assessed from biological process via the GO item enrichment analysis. *P* < 0.05 indicated statistically significant, representing significant enrichment.

### Cell Culture

Human osteosarcoma cell lines (SW1353, U2OS, HOS) and normal osteoblasts (hFOB1.19) were extracted from the cell bank of the Chinese Academy of Sciences (Shanghai). RPMI 1640 (Gibco, Gaithersburg, MD., United States) was cultured with 10% fetal bovine serum (HyClone, Logan, United States) and 1% penicillin/streptomycin (Gibco) in an incubator at 37°C and 5% CO_2_. All cells were identified by short tandem repeat (STR) and passaged less than 6 months after recovery.

### Preparation of Tissues

Twelve-pair glioma tissue and matched normal tissue were isolated from patients with glioma surgically removed at Minhang Hospital (Shanghai, China). No preoperative chemotherapy or radiotherapy were subjected to patients in this research. All studies were approved by The ethics committee of Minhang Hospital and observed with the Declaration of Helsinki. All the subjects received written informed consent.

### Transfection of Cells

The cells were seeded into 6-well plates 1 day prior to transfection. The second day, when cell confluency was around 70–80%, Lipofectamine 2000 and small interfering RNA were diluted in serum-free medium for 5 min. Then diluted reagents were gently mixed and maintained for another 20 min. Next, cells were mixed with the mixture and incubated for 4–6 h. After that, cells were harvested for further experiments. The sequences of siRNAs were as follows: si-CDCA4, GCAGCUUUGCCACAUGCUUTT; si-NC, UUCUCCGAACGUGUCACGUTT.

### Extraction and Quantation of RNA

Whole RNA was obtained from cultured cells and fresh tissue using Trizol Regent (Invitrogen, United States). cDNA was synthesized using PrimeScript RT Kit (Promega, Madison, Wisconsin, United States), and qRT-PCR was performed using SYBR premixed Ex-TAQ^TM^(Dalian, China) on ABI 7500 real-time PCR system (Foster City applied Biological Systems, United States). GAPDH was an internal control. Determination of relative gene expression 2^–ΔΔCt^ method.

### Cell Proliferation Assay

1 × 10^3^ cells were cultured overnight in 96-well plates. CCK-8 (Dojindo, Rockville, United States) was added to the above cells and incubated for 2 h. OD value of 450 nm was measured on an automatic label reader (Bio-RAD, Hercules, United States). Our data was derived from three independent experiments in triplicate.

### Transwell Migration Assay

2 × 10^5^ cells were suspended in serum-free medium and then placed in the superior compartment of 8 μm-well Transwells (BD Biosciences, San José, CA, United States). The lower compartment with 10% FBS was regarded as a chemical attractant. After cells incubating at 37°C for 2 days and staining for 15 min, 5 fields of view (× 200) were stochastically chosen under the microscope to calculate translocated cells.

### Statistical Analysis

SPSS19.0 statistical software package was executed to analyze statistics. The mean ± standard deviation represented the data, derived from three separate experiments. Student’s *t*-test and one-way variance were applied to analyze qRT-PCR data. Two-tailed paired Student’s *t*-test was used for comparison between groups. Survival curves of patients with different CDCA4 expression levels were plotted and compared using Kaplan-Meier method. *P* < 0.05 suggested significant difference.

## Results

### Identification of Genes Related to Aerobic Exercise

**FIGURE 1:**
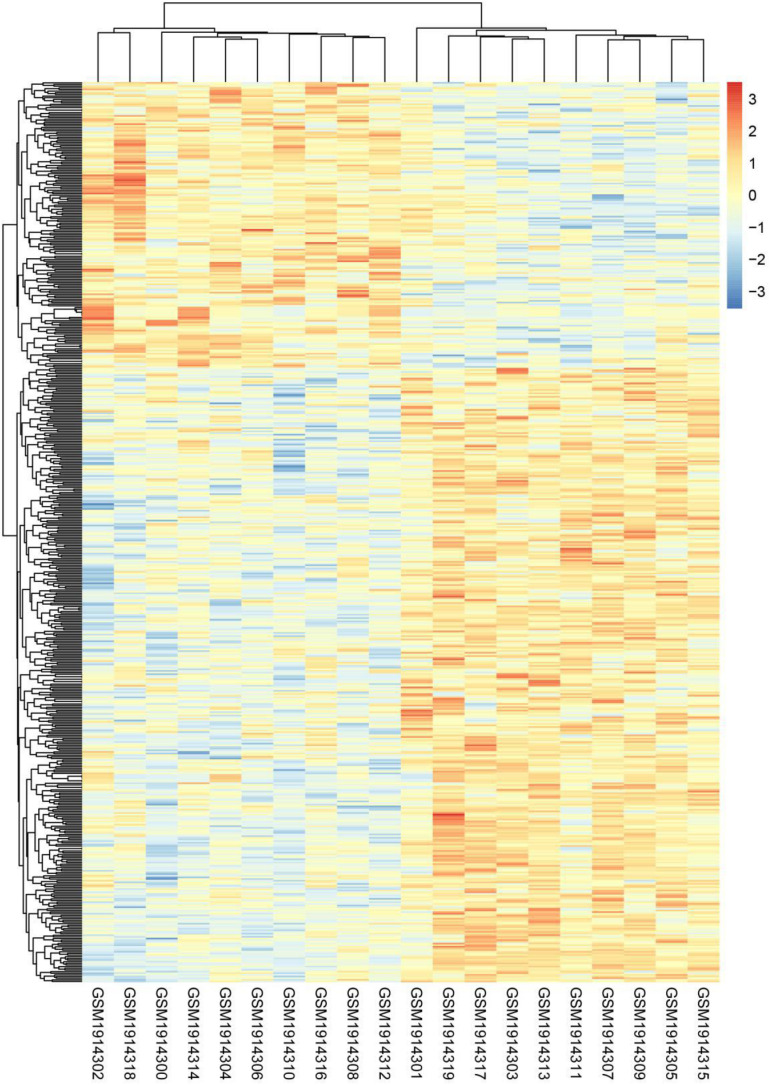
DEGs hierarchical cluster analysis between aerobic and anaerobic exercise groups in GSE74194 database. Three hundred seventy-three up-regulated DEGs related to aerobic exercise and 174 down-regulated DEGs related to aerobic exercise were identified. Red, up-regulated genes, blue, down-regulated genes.

Twenty subsets of transcriptome data were downloaded from the database GSE74194, including GSM1914302, GSM1914318, GSM1914300, GSM1914314, GSM1914304, GSM1914306, GSM1914310, GSM1914316, GSM1914308, GSM1914312, GSM1914301, GSM1914319, GSM1914317, GSM1914303, GSM1914313, GSM1914313, GSM1914305, and GSM1914315. The mRNA expression levels of the anaerobic and aerobic exercise groups were analyzed using the database. Heatmap analysis showed that there were 547 DEGs between the anaerobic exercise group and the aerobic exercise group, with 373 up-regulated genes and 174 down-regulated genes (Figure 1). Among them, CDCA4 was down-regulated.

### Analysis of PPI Network and Biological Processes

**FIGURE 2:**
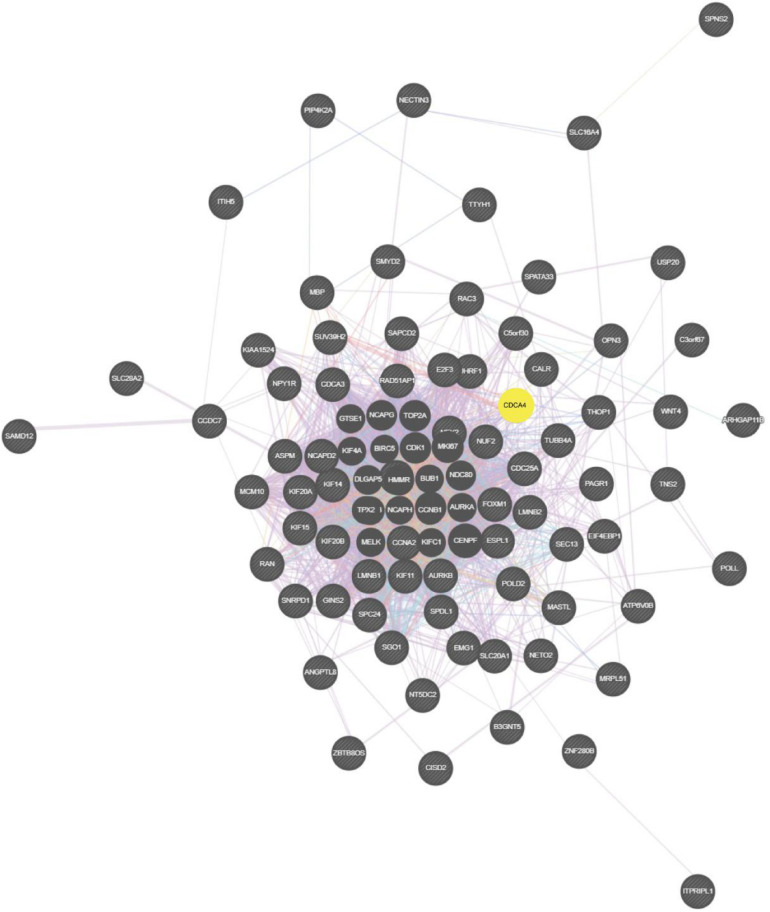
Totally, 90 aerobic exercise-related DEGs were included in PPI network. The nodes indicated proteins. The edges represented proteins’ interaction.

**FIGURE 3:**
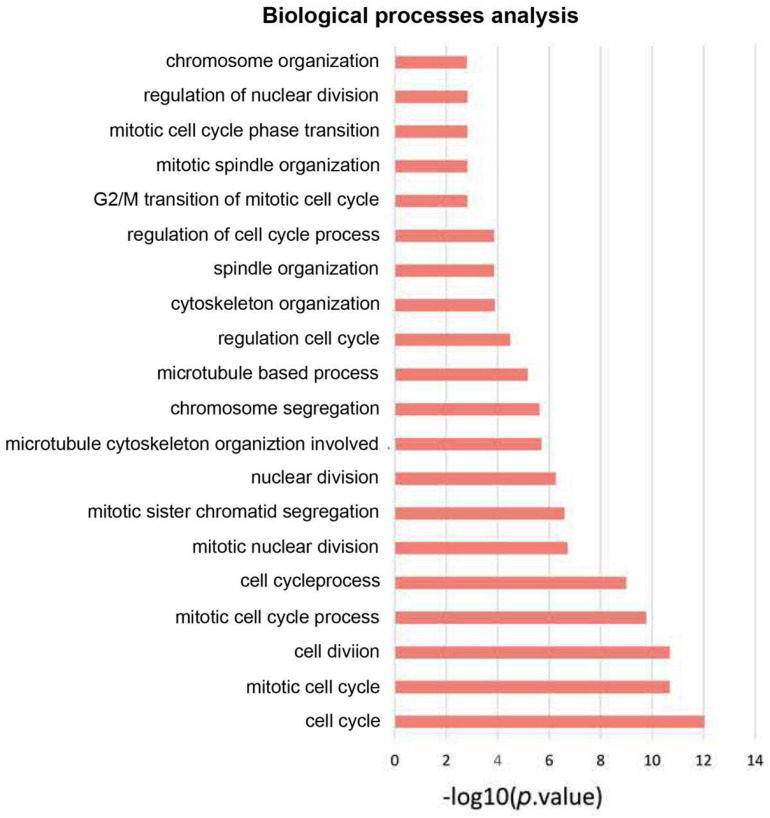
GO enrichment analysis of biological process of aerobic exercise-related DEGs with fold change >2.

On basis of the STRING database, Cytoscape software was performed to establish the DEGs PPI network. Ninety nodes were included in this network and probably displayed crucially in aerobic exercise (Figure 2). GO enrichment analysis of DEGs revealed that 20 terms were significantly enriched in biological processes, comprising mitotic cell cycle, cell division, mitotic nuclear division and sister chromatid segregation, nuclear division, microtubule cytoskeleton organization involved protein, microtubule-based process, spindle organization and G2/M transition of mitotic cell cycle (Figure 3).

### Highly Expressed CDCA4 in Osteosarcoma Patients Gave Rise to Poor Prognosis

**FIGURE 4:**
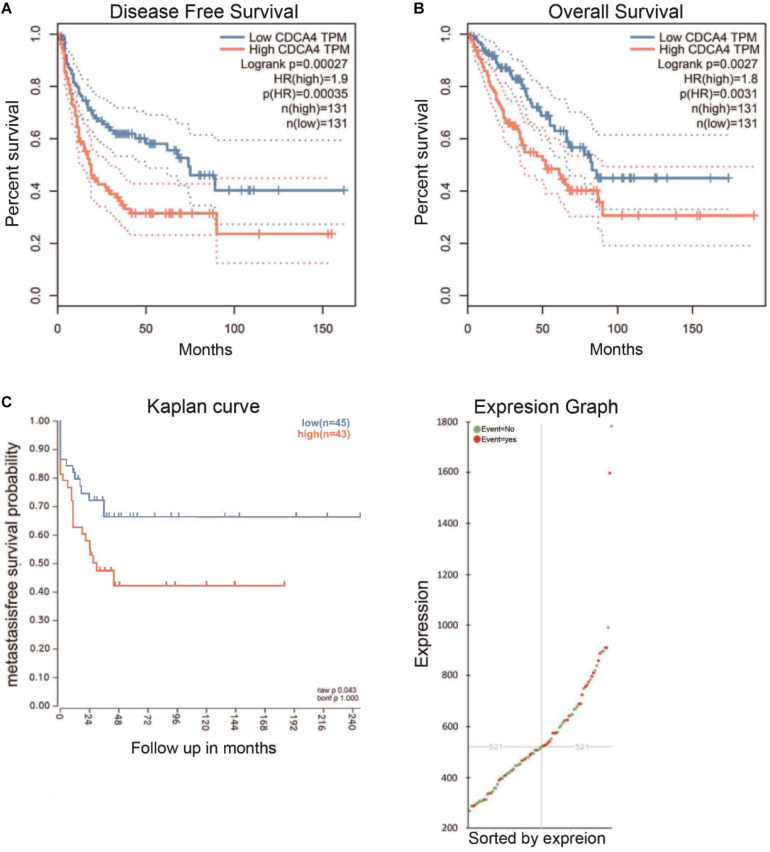
Kaplan Meier plotter online tool was conducted to identify the association between CDCA4 expression and the OS time **(A)**, disease-free survival **(B)**, and metastasis-free survival **(C)** of osteosarcoma patients. Patients with high levels of CDCA4 glioma had lower survival rates, and high levels of CDCA4 were negatively correlated with non-metastatic survival.

Kaplan-Meier survival analysis revealed that highly expressed CDCA4 in osteosarcoma patients led to shorter overall survival (OS) and disease free survival time (Figures 4A,B). In addition, patients with highly expressed CDCA4 displayed shorter metastasis-free survival (MFS), compared to those with lowly expressed CDCA4 (Figure 4C).

**FIGURE 5:**
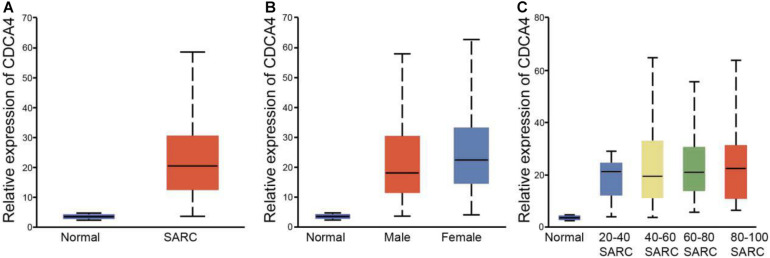
**(A)** According to the TCGA database, CDCA4 was highly expressed in SARC tissues. **(B)** CDCA4 expression level had correlation with the gender of SARC patients, and the expression was higher in female patients. **(C)** CDCA4 expression level displayed correlation with the age of SARC patients.

In order to explore whether CDCA4 expression was related to the clinicopathological features of osteosarcoma, we further analyzed the clinical data of osteosarcoma patients. Figure 5A showed that compared with normal tissues, CDCA4 expression in Sarcoma (SARC) tissues was dramatically higher. CDCA4 expression was correlated with gender (Figure 5B) and age (Figure 5C) in osteosarcoma patients.

### CDCA4 Expression Was Markedly Up-Regulated in Osteosarcoma Tissues and Cell Lines

**FIGURE 6:**
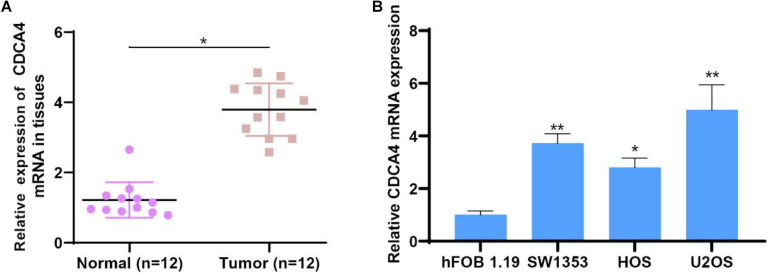
**(A)** CDCA4 was more highly expressed in osteosarcoma tumor tissues than normal tissues (*n* = 12). **(B)** CDCA4 was more highly expressed in cancer cells than normal cell, *P < 0.05, **P < 0.01.

To investigate CDCA4 functions in osteosarcoma, we measured CDCA4 expression in 12 paired normal and osteosarcoma tissues by qRT-PCR assays. The data indicated compared to normal tissue, CDCA4 expression level was largely raised in osteosarcoma tissues (Figure 6A). Next, we kept on evaluating CDCA4 expression in osteosarcoma cells (HOS, SW1353, and U2OS) and normal cell (hFOB1.19). The results showed that CDCA4 expression was greatly enhanced in osteosarcoma cells (Figure 6B).

### Reduction of CDCA4 Could Inhibit Osteosarcoma Cell Proliferation, Migration, and Invasion

**FIGURE 7:**
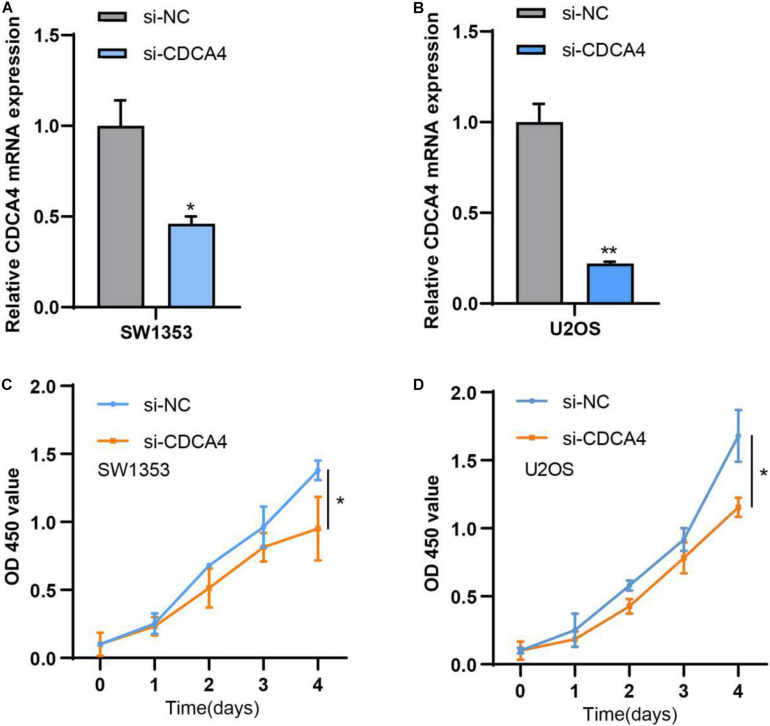
**(A)** CDCA4 expression was decreased in SW1353 after transfection of si-CDCA4. **(B)** CDCA4 expression was decreased in U2OS after transfection of si-CDCA4. **(C)** Reduced CDCA4 resulted in inhibited SW1353 cell proliferation. **(D)** Reduced CDCA4 resulted in inhibited U2OS cell proliferation,*P < 0.05, **P < 0.01.

**FIGURE 8:**
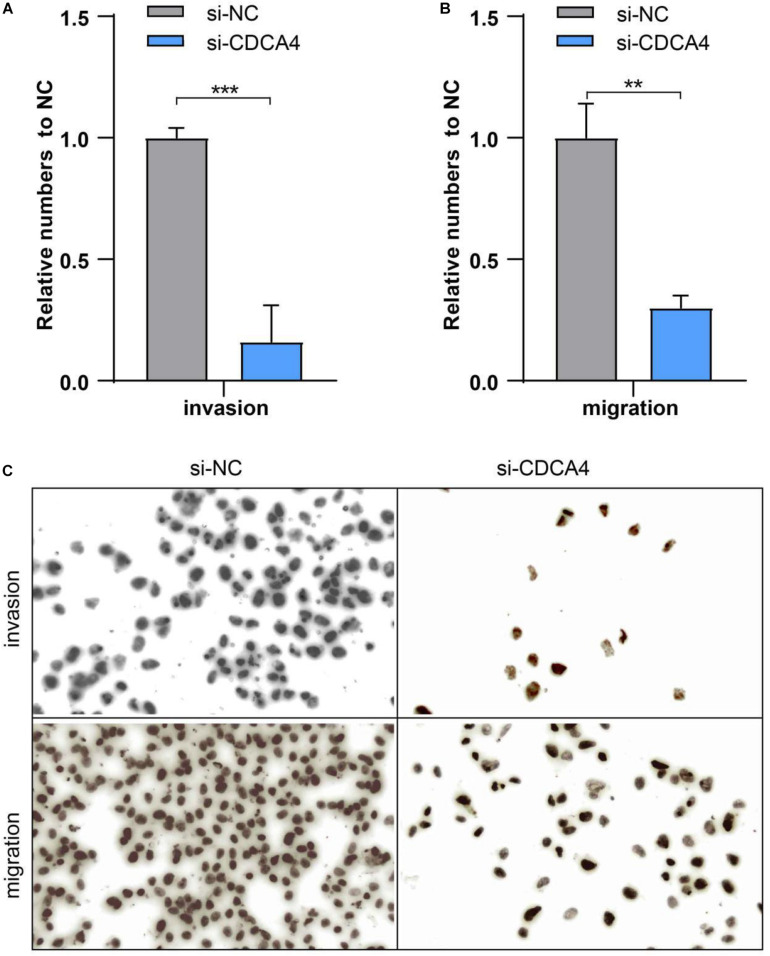
**(A)** Ablated CDCA4 obviously suppressed osteosarcoma cell invasion. **(B)** Ablated CDCA4 obviously suppressed osteosarcoma cell migration. **(C)** Detection of invasion and migration of osteosarcoma cells by Transwell assay, **P < 0.01, ***P < 0.001.

For clearly clarifying CDCA4 function in osteosarcoma occurrence and development, we transfected siRNA specifically targeting CDCA4 into SW1353 and U2OS cells, respectively. qRT-PCR data suggested that siRNA efficiently knocked down CDCA4 in SW1353 (Figure 7A) and U2OS (Figure 7B) cell. CCK 8 detection results revealed that reduced CDCA4 would suppress SW1353 and U2OS cell proliferation (Figures 7C,D). As shown in Figure 8, the results of Transwell assay showed that knocking out CDCA4 inhibited the invasion and migration of osteosarcoma cells. Taken together, our data indicated that reduction of CDCA4 could result in inhibition of osteosarcoma cell proliferation, migration, and invasion.

## Discussion

Aerobic exercise refers to a dynamic, rhythmic exercise involving a large number of muscle groups and aerobic metabolism in the body. Aerobic exercise can improve bone health and restore hemostasis of bone tissue by restoring bone biomarkers including bone alkaline phosphatase and calcium (Santos et al., 2017; Al Dahamsheh et al., 2019). Osteosarcoma is a malignant bone tumor consisting of mesenchymal cells that produce osteoid and immature bone. Successful treatment of osteosarcoma patients requires the close cooperation of an experienced multidisciplinary teams, including pediatric or internal oncologists, surgeons, pathologists, and radiologists (Kager et al., 2017; Xu et al., 2020). Physical exercise has been identified as a low-cost, safe and effective way to treat chronic intractable pain (Senba and Kami, 2017). Through the analysis of gene differential expression on the GSE74194 database, we obtained 547 aerobic exercise-related DEGs, 373 were up-regulated and 174 were down-regulated.

Many genes are reported to participate in osteosarcoma tumorigenesis (Cambien et al., 2008; Daino et al., 2009; Yang et al., 2020). Amid these genes, growth and differentiation factor 15 is one of representative gene. Its expression is increased in metastatic osteosarcoma tissues and has relation with the OS of osteosarcoma patients. Meanwhile, it is significantly related to the time of lung metastasis-free survival (MFS) (Chen et al., 2019). Yin et al. (2017) showed that the metastasis inhibitor gene KISS-1 modulated apoptosis and autophagy processes of osteosarcoma. However, the molecular mechanism of CDCA4 in osteosarcoma has not been clarified.

CDCA4 encodes a protein that belongs to the E2F family of transcription factors and is widely expressed in bone marrow. It can regulate the transcriptional activity of target genes like p53, E2F, JUN in the cell cycle and proliferation (Watanabe-Fukunaga et al., 2005; Hayashi et al., 2006; Tategu et al., 2008). CDCA4 functions importantly during many biological processes, such as proliferation and apoptosis (Hayashi et al., 2006; Xu et al., 2018). Although the research on the structure and function of CDCA4 has been gradually carried out, the depth and breadth still need to be expanded. In this study, 90 key genes related to aerobic exercise were obtained through PPI network analysis, including CDCA4, LMNB2, BUB1, TPX2, PAD51AP1, KIF4A, MEUK, SPDL1, RAN, and POLD2, etc. DAVID method was executed to perform Gene ontology analysis for biological processes (BP). The data revealed that DEGs modulated mitotic cell cycle, cell division, mitotic nuclear division and sister chromatid segregation, nuclear division, microtubule cytoskeleton organization involved protein, microtubule-based process, spindle organization, G2/M transition of mitotic cell cycle. GO enrichment analysis indicated that CDCA4 was largely enriched in cell cycle (*P* < 0.05). According to the results of the comprehensive analysis, CDCA4 was selected for further research.

In order to explore the role of CDCA4 in osteosarcoma, based on the TCGA database, CDCA4 expression, prognostic value and clinicopathological features in osteosarcoma were analyzed. Compared with normal tissue, CDCA4 expression was significantly increased in SARC tissues. The high CDCA4 level was associated with poor prognosis in SARC patients. In addition, in patients with SARC, CDCA4 expression was related to gender and age. Studies have found that osteosarcoma has a bimodal age distribution, with the first peak in adolescence and the second peak in adulthood (Ottaviani and Jaffe, 2009). The first peak is in the age range of 10–14 years, which coincides with the sudden increase in puberty growth. It is suggested that the sudden increase of osteosarcoma in adolescence is closely related to the occurrence of osteosarcoma. The second peak of osteosarcoma occurred in adults over 65 years old. A second malignancy is more likely to be associated with Paget’s disease (Gennari et al., 2019). It has always been believed that the incidence of osteosarcoma in men is higher than in women. Interestingly, the expression level of CDCA4 is higher in female SARC patients, which may be related to the patients’ aerobic exercise. Taken together, these findings indicated that CDCA4 was a potential novel target for the treatment of SARC patients and a biomarker for prognosis.

Subsequently, qRT-PCR analysis showed that CDCA4 was highly expression in osteosarcoma tissues and cell lines. *In vitro* functional test data showed that CDCA4 was closely related to the regulation of osteosarcoma cell proliferation, migration and invasion. In summary, our findings indicate that CDCA4 may play a carcinogenic role in osteogenic sarcoma. Interestingly, the analysis of the GSE74194 database found that the expression of CDCA4 decreased after aerobic exercise. The major objective of the exercise-oncology research is to evaluate how aerobic exercise affect the incidence, progression, and metastasis of cancer and relevant biological mechanisms. A number of observational evidence showed that higher levels of exercise were negatively associated with the incidence of several types of cancer (Campbell et al., 2007; Esser et al., 2009; Friedenreich et al., 2010; Jones and Alfano, 2013). Also, there are data showing that exercise after cancer diagnosis may improve prognosis of early-stage cancers such as prostate and colorectal cancer (Ballard-Barbash et al., 2012; Gueritat et al., 2014; Ashcraft et al., 2016). The current hypothesis is that exercise regulates tumor progression by regulating host-tumor interactions (Betof et al., 2013). Tumor progression is regulated by complex and multifaceted interactions between the host, tumor microenvironment and cancer cells which are subject to systemic and local growth factors, cytokines, hormones, and so on. Factors such as IL6,TNF, leptin, and insulin have already been reported to associate with higher recurrence and mortality (Betof et al., 2013). Obviously, manipulating these factors through exercise can influence cancer progression. Therefore, in our study, we guess that aerobic exercise can decrease the expression of oncogene CDCA4 to impede cancer development, indicating that appropriate aerobic exercise might be helpful to the treatment and prognosis of patients with osteosarcoma. All in all, these data suggested that aerobic exercise could affect osteosarcoma by regulating CDCA4 expression which provided a whole new perspective for the study of osteosarcoma.

To sum up, comprehensive bioinformatics analysis provides a simple and flexible method. Through a comprehensive analysis of the GSE74194 database, we have identified key genes related to aerobic exercise. CDCA4 expression was lower in aerobic exercise group than in anaerobic exercise group. Based on the analysis of the TCGA-SARC database, the upregulation of CDCA4 expression was associated with poor prognosis in patients with SARC. We conducted a series of experiments in the study to explore the exact role of CDCA4 in osteosarcoma progression. Our data showed that CDCA4 was elevated in osteosarcoma tissues and cell lines and affected cell proliferation, migration, and invasion. All in all, our research may provide a new perspective for the study of osteogenic sarcoma, indicating aerobic exercise can help improve the treatment of patients with osteogenic sarcoma.

## Data Availability Statement

The original contributions presented in the study are included in the article/supplementary material, further inquiries can be directed to the corresponding author/s.

## Ethics Statement

The studies involving human participants were reviewed and approved by the ethics committee of Minhang Hospital. The patients/participants provided their written informed consent to participate in this study.

## Author Contributions

SH, JZ, and XZ: conception and design. JQ, LY, and QX: development of methodology. SH, JZ, and JQ: analysis and interpretation of data. XZ, JQ, LY, and QX: writing, review, and revision of the manuscript. All authors contributed to the article and approved the submitted version.

## Conflict of Interest

SH was employed by the company Shuangwu Information Technical Company Ltd. The remaining authors declare that the research was conducted in the absence of any commercial or financial relationships that could be construed as a potential conflict of interest.

## Footnotes

1 https://www.ncbi.nlm.nih.gov/geo/query/acc.cgi

2 https://www.cancer.gov/about-nci/organization/ccg/research/structural-genomics/tcga

3 http://www.stringdb.org/

## Abbreviations

BP: Biological Processes
CDCA4: Cell Division Cycle-associated Protein 4
DEGs: Differentially Expressed Genes
GO: Gene Ontology
GEO: Gene Expression Omnibus
MFS: Metastasis-free Survival
NSCLC: Non-small Cell Lung Cancer
OS: Overall Survival
PPI: Protein-protein Interaction Network
qRT-PCR: Quantitative Reverse Transcription Polymerase Chain Reaction
STRING: The Search Tool for the Retrieval of Interacting Genes
SARC: Sarcoma
TNBC: Triple Negative Breast Cancer
TCGA: The Cancer Genome Atlas.
